# Homocysteine, blood pressure and gene–diet interactions in relation to vascular function measures of black South Africans

**DOI:** 10.1186/s12263-024-00751-8

**Published:** 2024-08-01

**Authors:** Jacomina P du Plessis, Leandi Lammertyn, Aletta E. Schutte, Cornelie Nienaber-Rousseau

**Affiliations:** 1 Centre of Excellence for Nutrition, North-West University, Private bag x6001, Box 594, Nutrition, Potchefstroom, 2520 South Africa; 2 Hypertension in Africa Research Team, North-West University, Potchefstroom, 2520 South Africa; 3 SAMRC Extramural Unit for Hypertension and Cardiovascular Disease, Faculty of Health Sciences, North- West University, Potchefstroom, 2520 South Africa; 4 School of Population Health, University of New South Wales, The George Institute of Global Health, Sydney, NSW Australia

**Keywords:** Precision nutrition, Vascular function, Gene-diet interactions, Hyperhomocysteinemia, H-type hypertension, Nutrigenetics, Hcy-polymorphisms

## Abstract

We investigated circulating homocysteine (Hcy), a cardiovascular disease (CVD) risk factor, examining its dietary associations to provide personalized nutrition advice. This study addressed the inadequacy of current dietary interventions to ultimately address the disproportionately high incidence of CVD in Black populations. Methods and Results: Cross-sectional analyses of 1,867 Black individuals of the PURE-SA study allowed the identification of dietary intake and cardiovascular measure interactions on three sub-categories: (1) normal blood pressure (BP), hypertension or Hcy-related hypertension (H-type), (2) low, normal or high Hcy concentrations, and (3) Hcy-related genetic combinations. Favorable body composition, but adverse dietary intake and cardiovascular determinants, were observed in higher Hcy categories. H-types, compared to regular hypertensives, had higher alcohol and lower macronutrient and micronutrient consumption. Inverse associations with carotid-radial pulse wave velocity were evident between monounsaturated fatty acid (FA) consumption and H-type hypertension as well as polyunsaturated FA and *CBS*883/ins68 TT carriers. Energy intake was positively associated with vascular cell adhesion molecule-1 (VCAM-1) in variant *CBS*T883C/ins68 and *CBS*9276 GG carriers. VCAM-1 was also positively associated with plant protein intake in *CBS*9276 GG and *MTR*2756 AA carriers and negatively with total protein intake and *CBS*9276 GG carriers. Alcohol intake was positively associated with intercellular adhesion molecule-1 in *MTR*2756 minor allele carriers. Conclusion: Because Hcy gene-diet interactions are evident, personalized nutrition, by adjusting diets based on genetic profiles (e.g., *CBS* and *MTR* variations) and dietary interactions (e.g., FAs and proteins), can enhance cardiovascular outcomes by managing Hcy and related hypertension in genetically susceptible individuals.

**Supplementary Information:**

The online version contains supplementary material available at 10.1186/s12263-024-00751-8.

## Introduction

Cardiovascular disease (CVD) is a leading cause of death in South Africa, with one in six deaths attributed to it [1]. High blood pressure (BP) is a primary risk factor for CVD. Sub-Saharan Africa has higher hypertension prevalence and lower BP control rates than other regions [2], with cardiovascular events occurring about 15 years earlier [3]. National surveys in South Africa reported hypertension rates of 38.4% in 2012 (SANHANES) and 48.2% in 2016 (DHS) [4].

Homocysteine (Hcy) is a nonessential amino acid in a vitamin-regulated, one-carbon methylation pathway. Elevated circulating Hcy concentrations – representing hyperhomocysteinemia (HHcy) – not only impact health adversely [5] but are associated with increased all-cause mortality risk, with each 5 µmol/l rise escalating rates by 34% [6]. HHcy is recognized as an independent risk factor for hypertension and CVD [7], inducing arterial wall structural and functional changes thereby influencing arterial stiffness [8]. However, data on HHcy’s effects among the Black South African population are scarce. Determining HHcy’s role in the heightened mortality, uncontrolled hypertension, and CVD in developing countries such as South Africa, also struggling with nutritional issues [9], is critical.

Diet is a modifier of Hcy concentrations. Determinants encompass protein, B vitamins, vegetables, fruits, omega 3 and 6 fatty acids, pulses, nuts, seeds and alcohol consumption, as previously reported [10–12]. A diet impacting Hcy through these determinants suggests potential disease-prevention strategies [13, 14]. Whether diet could be a possible means of influencing HHcy – and by extension cardiovascular function and inflammation – should be investigated while also considering genetic factors.

Several single nucleotide polymorphisms (SNPs) in the vitamin-regulated methylation pathway are relevant to Hcy. For our investigation, we included candidate SNPs known to impact this pathway. The methylenetetrahydrofolate reductase (*MTHFR*) C677T polymorphism (rs1801133) was selected because it is known to reduce enzyme activity, impairing methionine metabolism and leading to elevated Hcy concentrations. In addition, we included the cystathionine β synthase (*CBS*) T833C/844ins68 (rs5742905) and G9276A (a novel SNP) polymorphisms due to their roles in Hcy clearance. Variants in the *CBS* gene are critical for the transsulfuration pathway, which helps to lower Hcy concentrations by converting it to cystathionine. The methionine synthase (*MTR*) A2756G (rs1805087) polymorphism was also analyzed because it influences the remethylation pathway, promoting the conversion of Hcy to methionine and thus affecting Hcy concentrations.

The etiology of hypertension and CVD remains unclear, highlighting the need for research on Hcy-related genes and their interaction with environmental factors, especially diet, in developing these conditions. This focus is crucial, as diet is one of the few easily modifiable factors. The aim of this study was to advance the understanding of how Hcy interacts with CVD risk markers overall but also in respect of the genetic make-up of individuals with the benefit of precision nutrition. To this end, we used arterial stiffness as indicated by carotid-radialis pulse wave velocity (cr-PWV), endothelial activation [indicated by circulating intercellular adhesion molecule-1 (ICAM-1) and vascular cell adhesion molecule-1 (VCAM-1)] and, also, pulse pressure (PP), heart rate (HR), systolic (SBP), and diastolic BP (DBP) as outcome variables when investigating interactions with Hcy subdivisions and manifesting genotypes for *MTHFR* C677T, *MTR* A2756G and *CBS* T833C/844ins68 and G9276A. This work underscores the importance of including Hcy in hypertension screening and suggests personalized dietary advice for managing CVD risk among Black South Africans, considering individual Hcy concentrations, hypertension status and specific Hcy SNPs. This work highlights the potential of personalized dietary advice, based on individual Hcy concentrations and specific genetic profiles. This approach could possibly improve the management of chronic conditions, particularly CVD risk and improve overall health outcomes in populations vulnerable to hypertension and cardiovascular issues.

## Materials and methods

### Study design and participants

This cross-sectional study utilized baseline data from the South African arm of the Prospective Urban and Rural Epidemiology (PURE–SA) study, part of a multinational cohort across 17 countries investigating risk factors for chronic diseases in transitioning communities [15]. Following an eligibility screening of 6,000 homes, Tswana-speaking South Africans over 35 from specific North West province areas, deemed healthy and not pregnant or lactating, were randomly selected for participation. Volunteers were informed in the language of their choice and required to provide signed informed consent before any data were acquired. Participants could withdraw or refuse any measurement at any stage of the study without consequence. For this investigation, only individuals who had BP and Hcy data were included for analyses (*n* = 1,867). North-West University’s Health Research Ethics Committee approved the PURE–SA and sub-study protocols (ethics numbers: 04M10 and NWU-00142-18-A1), adhering to the Declaration of Helsinki principles, ensuring voluntary participation, anonymity and confidentiality.

### Questionnaires

Data on demographics and lifestyle were collected through interviews using standardized questionnaires by fieldworkers [15]. Food intake was assessed with quantitative food frequency questionnaires (QFFQs), validated against 7-day weighed food records and biomarkers [16], supplemented by food portion photograph books for accurate portion size reporting. QFFQ data were logged using FoodFinder3^®^ [South African Medical Research Council (SAMRC), Tygerberg, 2007] and analyzed for nutrients by the SAMRC. Dietary supplement use was negligible and excluded from micronutrient intake calculations.

### Anthropometric measures

Anthropometric measurements, including height (IP 1465, Invicta, London, UK), weight (Precision Health Scale, A&D Company, Tokyo, Japan), hip (HC) and waist circumference (WC) (Lufkin steel tape, Cooper Tools, Apex, NC, USA), were obtained with calibrated instruments following the International Society for the Advancement of Kinanthropometry (ISAK) guidelines [17]. Body mass index (BMI) and waist-to-hip ratio (WHR) were calculated from height, weight (kg/m^2^), and circumferences, respectively.

### Cardiovascular marker measurements

To ensure accuracy under standardized conditions [18], participants were instructed to avoid exercise, smoking, and caffeine use prior to measurements, and to observe a 5-minute rest. An Omron HEM-757 device (Omron Healthcare, Kyoto, Japan) measured brachial SBP, DBP, HR, and calculated PP in seated participants with their right arm at heart level. cr-PWV was assessed in a supine position on the left side using a Complior SP device (Artech-Medical, Pantin, France). Hypertension was diagnosed at BP ≥ 140/90 mmHg or with anti-hypertensive medication use [18, including thiazide diuretics, calcium channel blockers, and renin–angiotensin system inhibitors. H-type hypertension was identified in hypertensive individuals with Hcy levels ≥ 10 µmol/l [19].

### Laboratory biochemical measurements

Volunteers fasted overnight before a registered nurse collected blood samples from the antecubital vein before 11:00. Samples were centrifuged at 2,000 × g for 15 min to separate serum, plasma, and buffy coat, then stored at − 80 °C. Total plasma Hcy was measured using fluorescence polarization immunoassay [CV = 4.52%] on an Abbott immunoassay analyzer (AxSYM, Abbott Laboratories, Abbott Park, IL, USA). Sequential multiple analyzers from Konelab 20i (Thermo Scientific, Vantaa, Finland) and Cobas Integra 400 Plus (Roche, Basel, Switzerland) analyzed biomarkers including high-sensitivity C-reactive protein (hsCRP), gamma-glutamyl transferase (GGT) and all blood lipids [high-density lipoprotein cholesterol (HDL-c), total cholesterol (TC) and triglycerides], with low-density lipoprotein cholesterol (LDL-c) calculated via the Friedewald–Levy–Fredrickson formula [20]. Fasting plasma glucose was determined by the SynchronR System (Beckman Coulter Co., Fullerton, CA, USA) hexokinase method, and fasting glycated hemoglobin A1c (HbA1c) by the Bio-Rad Laboratories (Hercules, CA, USA) D-10 system. Sandwich ELISAs (human sICAM-1 and human sVCAM-1 assay, IBL, Hamburg, Germany) measured serum ICAM-1 and VCAM-1 levels. Whole blood tested for HIV with the First Response HIV 1-2-0 test (Premier Medical Corporation Ltd, Daman, India), confirmed by the Pareeshak card test (BHAT Bio-tech, Bengaluru, India) if positive. Participants on antiretroviral medication were not invited to partake, and those unaware of their HIV status were tested on the same day, ensuring unmedicated status in the analyzed variables even among seropositive individuals.

Genomic DNA was extracted from peripheral blood leukocytes using QIAGEN^®^, Flexigene^®^ DNA kits (QIAGEN^®^ Valencia, CA, USA; catalog number 51 206) and quantified by NanoDrop™ spectrophotometry (ND-1000, Wilmington, DE, USA). Five Hcy-related SNPs—*MTHFR* C677T (rs1801133), *CBS* T833C (rs5742905), *CBS* 844ins68 (indel, no rs#), *CBS* G9276A (novel SNP, no rs#) and *MTR* A2756G (rs1805087)—were genotyped using PCR and restriction fragment length polymorphism methods [21]. Sample DNA batches were analyzed with positive and negative controls to prevent misinterpretation from digestion issues or contamination. *MTHFR* and *MTR* genotypes were identified by analyzing enzyme-digested PCR fragments via electrophoresis on an ethidium bromide-stained 2% agarose gel. For *CBS* alterations, both undigested and digested PCR products were examined. Two independent researchers, blinded to the Hcy phenotype, scored the genotypes and merged their results. Discrepancies were resolved by reanalyzing the genotypes. For primers and digestion enzymes used, see Supplementary Table 1.

### Statistical analysis

Statistica version 14.0 (TIBCO Software Inc., Tulsa, OK, USA) and R version 4.2.0 (R Foundation for Statistical Computing, Vienna, Austria; 2020) were used for data analysis. Shapiro–Wilk W and Kolmogorov–Smirnov tests assessed normality. Descriptive statistics for non-normal data were presented as medians and interquartile ranges, and for normal data as means ± standard deviations. Normal and partial Spearman rank correlations (adjusting for age, WHR, HDL-c, and GGT) assessed Hcy’s relationship with dietary intake. Mann–Whitney U tests compared dependent continuous variables across three groups (normal BP vs. hypertension, normal BP vs. H-type hypertension, hypertension vs. H-type hypertension). General linear models (GLMs) identified differences among low (< 7.0 µmol/l), normal (7.0–12.0 µmol/l), and high (> 12.0 µmol/l) Hcy categories [22].

To explore associations between CVD markers, dietary components, Hcy categories, BP categories, and Hcy-related genetic polymorphisms, factorial GLMs, adjusted for age, assessed interaction effects. The *CBS* T833C and *CBS* 844ins68 SNPs, being in complete linkage, were reported as *CBS* T833C/844ins68 [21]. Heterozygotes and homozygotes for variant alleles were combined following the dominant genetic mode. Partial Spearman correlations, adjusted for age and added sugar, analyzed relationships between CVD markers, dietary intake, BP sub-groups, Hcy sub-groups, and genotypes using this genetic coding.

For general analyses, *P* ≤ 0.05 indicated statistical significance. To address multiple testing and false discovery rates, Hochberg and Benjamini’s method was applied. With a false discovery rate of 25%, considering 4 SNPs (*CBS* 833 SNP linked with 844ins68), subdivisions as 1, dietary intake categories as 5, and cardiovascular markers as 1 due to associations with dietary factors, the significance threshold was set at *P* < 0.01 [1/((4 + 1)51))(0.25) = 0.01].

## Results

### Characteristics compared across Hcy sub-groups and correlations with Hcy

The characteristics and differences across Hcy sub-groups are detailed in Supplementary Table 2. Most of the recruits presented with normal Hcy concentrations (56.7%), whereas 18.3% demonstrated low and 25.0% high levels. Age, HDL-c, and GGT increased over rising Hcy sub-groups. Furthermore, certain body composition variables were more favorable (weight, BMI, and WC decreased; HC and WHR increased) over sub-groups with increasing Hcy concentrations, these associations attenuated after adjusting for sex. A quarter of HIV-seroposiive individuals were in the low-Hcy category, whereas a quarter of those without the virus presented with high Hcy, resulting in frequency differences among the Hcy sub-groups. However, Hcy concentrations did not differ between those with or without HIV (*P* = 0.99). Alcohol intake was greater in the high-Hcy category (17.2 g/d) – more than double that of the low-Hcy category intake (8.14 g/d); this association remained after adjustments. Energy intake, total protein, animal protein, added sugar, and fat intake (including SFAs, MUFAs, PUFAs, omega-6 FAs, omega-3 FAs and cholesterol) were lower in the highest-Hcy category. Fruit and vegetable intake and B vitamins (biotin, pantothenic acid, vitamin B_6_, and vitamin B_12_) were also lowest in the high-Hcy group. For biotin and vitamin B_6_, those presenting with normal and high Hcy and for vitamin B_12_ those with high Hcy, respectively, did not meet the recommended daily allowances (RDAs) of 30 µg, 1.3–1.5 mg and 2.4 µg, respectively. For pantothenic acid, none of the Hcy sub-groups met the RDA of 5 mg, with the intake values declining over increasing Hcy sub-groups. Even though folate, niacin and riboflavin intake ranges did not differ among the Hcy sub-groups, no sub-group’s intake met the RDA of 400 µg, 1.1–1.3 mg and 14.0–16.0 mg, respectively, and tended to be lower over increasing Hcy sub-groups. Of the cardiovascular measures, SBP, DBP, PP, HR, and cr-PWV differed among the Hcy sub-groups, being higher in the HHcy sub-group with DBP and cr-PWV exceeding normal healthy ranges [18, 23].

Supplementary Table 3 shows correlations between Hcy and various markers. Initially, Hcy positively correlated with age, WHR, HDL-c, GGT, some cardiovascular markers, and alcohol intake (all *r* ≥ 0.15), and negatively with most dietary intake markers (*r* ≤ 0.15). In model 2, after adjusting for age, WHR, HDL-c, and GGT, excluding collinear variables, positive correlations remained for age, HDL-c, GGT and added sugar’s percentage of total carbohydrate intake (all *r* ≥ 0.15). Post-adjustment, only Hcy and HR showed a weak correlation among cardiovascular markers.

### Daily dietary intake compared among blood pressure sub-groups

To explore the association between Hcy as well as its dietary determinants and BP as a cardiovascular risk factor, sub-groups for blood pressure (BP) were created including a group for H-type hypertension; differences are noted in Table 1. While there is an age difference between normal BP and regular hypertension groups, it is not present between normal BP and H-type hypertension. Those with regular hypertension also presented with B-vitamin intake ranges meeting the RDAs except for folate and pantothenic acid, whereas those with H-type hypertension did not satisfy the RDAs except for thiamin and vitamin B_12_. The difference between the hypertension vs. H-type hypertension groups’ energy intake was not attributed to age, weight, GGT, alcohol, added sugar and carbohydrates alone or combined, but due to differences in either total protein or fat intake. Genotype frequency differences among BP subgroups are detailed in another study [24].

**Table 1:** Daily dietary intake of participants compared among blood pressure sub-groups

Dietary intake variables		Median (25th − 75th ) or mean ± SD or *n* (%)			*p* value for difference among BP sub-groups (Mann–Whitney U)		
		Normal (*n* = 1,044)	Hypertension* (*n* = 526)	H-type hypertension* (*n* = 425)	Normal vs. HTN	Normal vs. H-type HTN	HTN vs. H-type HTN
Age (years)		45.0 (40.0–53.0)	49.0 (43.0–57.0)	53.0 (46.0–61.0)	< 0.0001	< 0.0001	< 0.0001
Sex, n (%)	Male	394 (52.9)	161 (21.6)	190 (25.5)	< 0.05	< 0.05	< 0.05
	Female	652 (52.1)	365 (29.1)	235 (18.8)	< 0.05	< 0.05	< 0.05
Urbanization level, n (%)	Urban	457 (45.7)	332 (33.2)	210 (21.1)	< 0.05	< 0.05	< 0.05
	Rural	589 (59.0)	194 (19.5)	215 (21.5)	< 0.05	< 0.05	< 0.05
Tobacco use, n (%)	Current	544 (52.7)	273 (26.4)	217 (20.9)	< 0.05	< 0.05	< 0.05
	Former	39 (51.3)	18 (23.7)	19 (25.0)	< 0.05	< 0.05	< 0.05
	Never	457 (52.1)	231 (26.3)	189 (21.6)	< 0.05	< 0.05	< 0.05
BMI (kg/m^2^)		22.1 (18.9–27.5)	25.3 (20.2–31.6)	23.1 (19.5–28.8)	< 0.0001	0.01	0.003
Energy (kJ)		6,933 (5,111–9,471)	7,833 (5,702–11,226)	7,065 (5,248–9,893)	0.0001	0.53	< 0.001
Alcohol (g)		10.8 ± 23.5	11.0 ± 21.9	15.0 ± 25.6	< 0.0001	< 0.0001	0.01
Protein (%TE)		11.5 (10.3–12.8)	11.5 (10.3–12.8)	11.6 (10.4–12.9)	< 0.01	0.88	0.07
Protein (g)		46.7 (33.9–66.1)	55.0 (37.9–82.0)	48.0 (34.4–68.4)	< 0.0001	0.62	< 0.0001
Plant protein (g)		28.5 (20.4–37.6)	30.5 (22.2–41.5)	28.0 (20.2–38.2)	0.04	0.62	0.01
Animal protein (g)		16.4 (9.01–28.6)	23.5 (12.6–35.3)	18.1 (10.6–28.6)	< 0.0001	0.80	< 0.0001
Carbohydrate (%TE)		61.5 (54.9–68.7)	58.5 (53.7–65.3)	60.1 (53.7–66.7)	< 0.0001	0.06	0.22
Added sugar (%TCHO)		15.3 (9.59–21.8)	15.6 (10.0–22.7)	14.3 (8.83–20.2)	0.49	< 0.01	< 0.01
Total fat (%TE)		21.9 (17.2–27.5)	23.8 (18.9–28.8)	22.0 (17.2–27)	0.01	0.35	< 0.001
SFAs (%TE)		5.10 (3.43–7.00)	5.71 (4.16–7.25)	5.16 (3.57–7.01)	0.01	0.89	< 0.01
MUFAs (%TE)		5.43 (3.56–7.61)	6.38 (4.33–8.18)	5.60 (3.72–7.55)	< 0.001	0.74	< 0.001
PUFAs (%TE)		6.73 (4.90–8.66)	7.19 (5.40–8.73)	6.60 (5.06–8.32)	0.25	0.20	< 0.01
Omega-6 FAs (g)		11.5 (10.3–12.8)	14.4 (8.71–21.9)	12.1 (10.4–12.9)	< 0.0001	0.21	< 0.0001
Omega-3 FAs (g)		0.32 (0.18–0.49)	0.39 (0.24–0.59)	0.32 (0.20–0.49)	< 0.0001	0.63	< 0.0001
Cholesterol (mg)		136 (74.0–244)	190 (94.4–302)	147 (81.0–246)	< 0.0001	0.93	0.0001
Dietary folate (µg)		353 (250–468)	372 (256–523)	344 (227–477)	0.22	0.16	< 0.01
Dietary vitamin B_1_ (mg) (thiamine)		1.48 (1.07–1.98)	1.53 (1.12–2.29)	1.47 (1.09–2.07)	0.13	0.57	0.07
Dietary vitamin B_2_ (mg) (riboflavin)		0.94 (0.63–1.50)	1.19 (0.71–1.80)	1.01 (0.67–1.62)	< 0.0001	0.33	0.02
Dietary biotin (µg)		28.4 (18.7–43.6)	36.4 (20.8–52.8)	29.4 (18.2–43.5)	< 0.001	0.36	< 0.0001
Dietary pantothenic acid (mg)		3.13 (2.11–4.71)	4.01 (2.44–5.65)	3.11 (2.20–4.75)	< 0.0001	0.32	< 0.0001
Dietary vitamin B_3_ (mg) (niacin)		12.0 (8.47–17.6)	14.2 (9.93–20.9)	12.6 (8.92–18.5)	< 0.0001	0.98	0.001
Dietary vitamin B_6_ (mg)		1.26 (0.89–1.75)	1.42 (0.94–2.12)	1.27 (0.91–1.75)	0.02	0.25	0.001
Dietary vitamin B_12_ (µg)		2.49 (1.12–4.77)	3.28 (1.47–6.04)	2.55 (1.28–4.94)	< 0.01	0.90	< 0.01
Fruit and vegetables (g)		80.1 (48.9–138)	104 (61.9–191)	86.4 (50.6–148)	< 0.0001	0.69	< 0.0001
Pulses, nuts and seeds (g)		2.93 (0.00–18.9)	5.71 (0.00–21.4)	2.86 (0.00–17.1)	0.17	0.32	0.02

FA, fatty acid; MUFAs, monounsaturated fatty acids; %TE, percentage total energy; %TCHO, percentage energy from carbohydrates; PUFAs, polyunsaturated fatty acids; SFAs, saturated fatty acids; TC, total cholesterol

*H-type hypertension is a subset of those presenting with both hypertension and Hcy > 10.0 µmol/l and has thus been excluded from the hypertension group

### Interactions with dietary intake in relation to cardiovascular markers

In Tables 2, 3 and 4 the interactions (*P* ≤ 0.01) between the BP sub-groups, Hcy sub-groups or Hcy-related SNPs and dietary intake are presented in relation to cardiovascular markers.

**Table 2:** Blood pressure category interactions with markers of nutritional status in relation to markers of cardiovascular function

Interactions with BP categories	Interaction *p* value	Genetic alleles	Unadjusted		Adjusted for age		Adjusted for age and added sugar	
			*r*	*p*	*r*	*p*	*r*	*p*
**in relation to SBP**								
Cholesterol	< 0.01	Normal BP	0.08	0.01	0.08	0.14	0.09	0.12
		HTN	0.08	0.06	–0.04	0.70	–0.02	0.83
		H-type HTN	0.02	0.70	–0.14	0.22	–0.16	0.18
Dietary vitamin B_12_	< 0.01	Normal BP	0.05	0.09	0.08	0.15	0.09	0.13
		HTN	0.05	0.26	–0.06	0.30	–0.04	0.69
		H-type HTN	0.05	0.26	–0.16	0.17	–0.17	0.14
**in relation to DBP**								
Dietary vitamin B_12_	0.01	Normal BP	0.01	0.78	0.05	0.41	0.05	0.40
		HTN	0.10	0.02	0.04	0.67	0.06	0.57
		H-type HTN	< 0.01	0.93	–0.10	0.41	–0.11	0.34
**in relation to PP**								
Protein	< 0.001	Normal BP	0.03	0.32	–0.06	0.28	–0.07	0.20
		HTN	0.15	< 0.001	–0.03	0.72	0.04	0.72
		H-type HTN	0.13	< 0.01	0.04	0.71	0.03	0.77
Animal protein	0.001	Normal BP	0.04	0.25	–0.05	0.40	–0.04	0.47
		HTN	0.09	0.05	–0.06	0.56	–0.05	0.61
		H-type HTN	0.12	0.01	0.04	0.75	0.04	0.71
**in relation to cr-PWV**								
Animal protein	0.01	Normal BP	–0.10	< 0.01	–0.05	0.42	–0.04	0.49
		HTN	–0.02	0.63	< 0.01	0.98	0.04	0.69
		H-type HTN	–0.05	0.34	0.02	0.84	0.03	0.80
Cholesterol	0.01	Normal BP	–0.06	0.05	0.01	0.92	0.01	0.84
		HTN	–0.02	0.66	0.02	0.83	0.05	0.60
		H-type HTN	–0.03	0.50	0.08	0.48	0.10	0.41
SFAs	0.001	Normal BP	–0.11	< 0.001	–0.11	0.05	–0.10	0.09
		HTN	–0.11	0.01	–0.07	0.50	–0.02	0.86
		H-type HTN	–0.12	0.01	–0.04	0.75	–0.06	0.63
MUFAs	0.01	Normal BP	–0.12	< 0.001	–0.12	0.03	–0.11	0.05
		HTN	–0.09	0.05	–0.08	0.46	–0.03	0.79
		H-type HTN	–0.10	0.03	0.01	0.93	–0.01	0.96
Carbohydrate	< 0.01	Normal BP	–0.02	0.59	–0.04	0.54	–0.03	0.63
		HTN	–0.01	0.86	–0.04	0.72	–0.05	0.65
		H-type HTN	–0.03	0.51	0.05	0.69	0.02	0.85

HTN, hypertension; MUFAs, monounsaturated fatty acids; %TE, percentage of total energy intake; SFAs, saturated fatty acids

*p* values for interaction obtained via GLM adjusted for age. First *r* and *P* value are for unadjusted Spearman correlations; second *r* and *p* value are adjusted for age; third additionally adjusted for added sugar

**Table 3:** Hcy categories interactions with markers of nutritional status in relation to markers of cardiovascular function

Interactions with Hcy categories	Interaction *P* value	Hcy category	Unadjusted		Adjusted for age		Adjusted for age and added sugar	
			*r*	*p*	*r*	*p*	*r*	*p*
**in relation to ICAM-1**								
Dietary biotin	< 0.01	Low	–0.10	0.27	–0.11	0.27	–0.11	0.28
		Normal	0.10	0.09	0.15	0.01	0.15	0.01
		High	0.10	0.28	0.15	0.14	0.16	0.11
Fruit and vegetables	< 0.01	Low	–0.04	0.71	–0.04	0.71	–0.04	0.71
		Normal	0.13	0.02	0.13	0.03	0.13	0.03
		High	–0.04	0.70	–0.02	0.86	0.01	0.94
**in relation to VCAM-1**								
Fruit and vegetables	< 0.01	Low	0.08	0.39	0.06	0.32	0.10	0.31
		Normal	0.08	0.16	0.07	0.26	0.07	0.22
		High	–0.03	0.75	0.003	0.97	0.01	0.93

*P* values for interactions obtained *via* GLM adjusted for age. First *r* and *P* value are for unadjusted Spearman correlations; second *r* and *P* value are adjusted for age; third additionally adjusted for added sugar

**Table 4:** *MTHFR*,* MTR* and *CBS* gene polymorphisms’ interactions with markers of nutritional status in relation to markers of cardiovascular function

	Interaction *p* value	Genetic alleles	Unadjusted		Adjusted for age		Adjusted for age and added sugar	
			*r*	*p*	*r*	*p*	*r*	*p*
**Interactions with ** ***MTHFR C677T***								
**in relation to cr-PWV**								
Added sugar	< 0.01	CC	–0.11	< 0.0001	–0.06	0.26	–	–
		CT/TT	–0.15	< 0.01	–0.02	0.87	–	–
**in relation to VCAM-1**								
Dietary vitamin B_12_	0.01	CC	0.02	0.73	–0.001	0.97	–0.001	0.98
		CT/TT	0.05	0.60	–0.05	0.68	–0.05	0.69
**Interactions with ** ***CBS T883C/ins68***								
**in relation to cr-PWV**								
Plant protein intake	0.01	TT	0.06	0.07	0.13	0.05	0.09	0.15
		TC/CC	–0.01	0.74	–0.03	0.67	–0.07	0.28
Dietary vitamin B_1_ (thiamine)	< 0.01	TT	0.08	0.01	0.14	0.02	0.11	0.10
		TC/CC	–0.04	0.70	–0.01	0.91	–0.05	0.43
Dietary vitamin B_6_	0.01	TT	0.04	0.26	0.09	0.15	0.07	0.27
		TC/CC	–0.05	0.16	–0.03	0.63	–0.07	0.32
PUFAs	0.01	TT	–0.13	< 0.0001	–0.15	0.01	–0.16	0.01
		TC/CC	–0.04	0.17	–0.04	0.52	–0.04	0.58
**in relation to VCAM-1**								
Energy intake	0.01	TT	0.06	0.29	0.06	0.35	0.06	0.33
		TC/CC	0.14	0.02	0.14	0.02	0.15	0.01
Protein	0.01	TT	–0.05	0.40	–0.07	0.27	–0.08	0.21
		TC/CC	–0.06	0.30	–0.06	0.32	–0.06	0.38
Animal protein	< 0.01	TT	–0.11	0.07	–0.12	0.05	–0.10	0.12
		TC/CC	–0.07	0.24	–0.09	0.13	–0.10	0.11
Dietary vitamin B_3_ (niacin)	0.01	TT	0.06	0.35	0.06	0.32	0.06	0.37
		TC/CC	0.10	0.11	0.10	0.11	0.12	0.06
Dietary pantothenic acid	0.01	TT	0.003	0.96	–0.01	0.32	< 0.001	0.99
		TC/CC	0.10	0.10	0.10	0.14	0.10	0.11
Omega-3 FAs	< 0.01	TT	0.002	0.97	–0.01	0.93	0.01	0.89
		TC/CC	0.05	0.37	0.06	0.34	0.06	0.35
**Interactions with ** ***CBS G9276A***								
**in relation to VCAM-1**								
Energy	0.01	GG	–0.03	0.35	0.15	0.01	0.14	0.02
		GA/AA	–0.01	0.86	0.03	0.63	0.03	0.63
Plant protein	< 0.01	GG	0.04	0.28	0.21	< 0.001	0.21	< 0.0001
		GA/AA	0.05	0.16	0.05	0.49	0.04	0.53
Dietary vitamin B_3_ (niacin)	0.01	GG	0.06	0.10	0.12	0.05	0.11	0.06
		GA/AA	0.02	0.53	0.02	0.79	0.02	0.82
**Interactions with ** ***MTR A2756G***								
**in relation to cr-PWV**								
SFAs	0.01	AA	–0.11	0.0001	–0.10	0.07	–0.11	0.05
		AG/GG	–0.06	0.15	–0.06	0.41	–0.02	0.75
**in relation to ICAM-1**								
Alcohol intake	< 0.01	AA	0.10	0.08	0.10	0.07	0.09	0.11
		AG/GG	0.12	0.10	0.15	0.05	0.15	0.04
**in relation to VCAM-1**								
Energy	< 0.01	AA	0.09	0.09	0.08	0.15	0.08	0.18
		AG/GG	0.12	0.09	0.12	0.12	0.12	0.12
Total protein	< 0.01	AA	–0.10	0.08	–0.12	0.04	–0.12	0.03
		AG/GG	–0.02	0.83	–0.02	0.80	–0.01	0.85
Plant protein	< 0.01	AA	0.11	0.04	0.15	< 0.0.1	0.14	0.01
		AG/GG	–0.001	0.99	0.13	0.09	0.14	0.07
Dietary vitamin B_6_	< 0.01	AA	0.12	0.03	0.11	0.05	0.10	0.07
		AG/GG	0.13	0.06	0.15	0.04	0.15	0.04
Dietary folate	0.01	AA	0.15	< 0.01	0.15	< 0.01	0.14	0.01
		AG/GG	0.18	0.01	0.20	< 0.01	0.21	< 0.01

FAs, fatty acids; %TCHO, percentage of total carbohydrate energy; %TE, percentage total energy; PUFAs, polyunsaturated fatty acids; SFAs, saturated fatty acids

*P* values for interactions obtained *via* GLM adjusted for age, but the interaction with cr-PWV is additionally adjusted for mean arterial pressure. First *r* and *P* values are for unadjusted Spearman correlations; second *r* and *P* values are adjusted for age, third additionally adjusted for added sugar

#### BP sub-group interactions

For those within the normal BP range, cholesterol intake had a positive relationship with SBP. Furthermore, dietary vitamin B_12_ intake had a protective inverse relationship with DBP in those with hypertension. Total protein, as a percentage of energy, linearly associated with PP in hypertensives and H-type hypertensives; animal protein showed a stronger positive correlation with PP in H-type hypertensives than in other BP categories, but inversely with cr-PWV in normal BP individuals. Consumption of SFAs showed negative associations with cr-PWV across all BP subdivisions, with H-type hypertension manifesting a slightly stronger correlation. MUFAs intake also displayed a negative linear association between cr-PWV in those with normal BP and H-type hypertension, and borderline associations with the hypertension groups as well. All correlations with BP sub-groups, except those with MUFAs intake in normotensives, became inconsequential after adjusting for age; after additionally adjusting for added sugar intake, this association became borderline as well.

#### Hcy sub-group interactions

Those presenting with Hcy concentrations within the normal ranges had a borderline positive association with biotin and a positive association between fruit and vegetable intake in relation to ICAM-1, whereas no association was observed in the other sub-groups. Partial Spearman correlations, after adjusting for age and then age plus added sugar, revealed stronger biotin associations and consistent positive relations with fruit/vegetable intake. Yet, significance vanished after adjusting interactions for added sugar.

#### Hcy-related polymorphism interactions

Added sugar displayed a nuanced relationship with cr-PWV among *MTHFR* genotypes, showing a stronger negative association in variant allele carriers compared to wildtype homozygotes at the 677 locus. Adjusting for fruit/vegetable intake and age nullified the significance.

*CBS* T883C/ins68 TT genotype carriers showed a notable inverse correlation with cr-PWV and PUFAs intake, not altered by age or added sugar, strengthening post-adjustment. In individuals with the TT genotype of *CBS* T883C/ins68, there was a positive correlation between thiamine intake and cr-PWV, while no significant correlation was observed in individuals with other genotypes After adjusting for added sugar, the interaction and sub-group correlations with thiamine were no longer significant. In those harboring the variant allele of *CBS* T883C/ins68, there was a positive linear association, even after additional adjustment, between energy consumption and VCAM-1, whereas in those harboring the wildtype allele no association was observed. The homozygote major G allele carriers of the *CBS* 9276 genotype also had strong positive correlations between VCAM-1 and energy as well as plant protein consumption only after adjusting for age and added sugar intake.

For *MTR* A2756G homozygote AA genotypes, a negative linear association between cr-PWV and SFAs intake persisted after adjusting for fruit and vegetable intake but disappeared with PUFAs adjustment. Age and added sugar adjustments further attenuated correlations. The minor G allele carriers exhibited a positive association between ICAM-1 and alcohol intake only after adjusting for age and added sugar. Total, plant proteins, vitamin B_6_, and folate intake positively correlated with VCAM-1 in AA genotypes. For heterozygotes and GG carriers, folate showed the strongest positive correlation with VCAM-1. Post-adjustment for age and added sugar, protein and folate associations persisted, while vitamin B_6_’s became borderline.

## Discussion

In this study we considered diet interactions across three sub-groups: (1) normal BP, hypertension, and Hcy-related hypertension (H-type); (2) low, normal, and high Hcy levels; and (3) various Hcy-related genotypes. We observed that those with HHcy expressed more favorable energy and macronutrient intake and body composition measures than those with normal and low-Hcy concentrations. However, hyperhomocysteinemic individuals reported noticeably higher alcohol and lower fruit and vegetable intake, possibly contributing to their inadequate vitamin status and higher CVD measures. There were more men than women in the HHcy category and, after accounting for sex, the body composition associations disappeared, although differences in alcohol and diet intake remained noteworthy. Moreover, 9 in 10 of those with HHcy also presented with hypertension (H-type) and when compared with the regular hypertension sub-group, H-type hypertensives recorded higher alcohol intake and lower fruit and vegetable consumption, including lower folate and other B vitamins intake, than those with hypertension alone. To complement our investigation, we report below on specific dietary aspects of BP categories, Hcy categories and Hcy-related genetic characteristics that associate with cardiovascular functional measures and CVD risk in a population already at a high risk.

### Blood pressure categories, dietary intake and cardiovascular function markers

BP measurement, an affordable, non-invasive tool, helps healthcare professionals detect and manage chronic conditions and is a step that could complement personalized nutrition. Yet, guidelines overlook H-type hypertension, which could significantly impact hypertension management and precision nutrition. We advise everyone, particularly individuals with hypertension, to consume adequate vitamin B_12_. Research on the direct effects of vitamin B_12_ intake and BP are scarce [25]; the most plausible link is through the Hcy folate–vitamin B_12_ dependent and independent remethylation metabolism, in which a reduced vitamin B_12_ intake results in higher Hcy concentrations and thereby raised hypertension and CVD risk.

We also advise individuals with regular and H-type hypertension to monitor their total protein intake, as it may raise PP, especially advising caution with animal protein for those with H-type, given PP’s significance in CVD risk [26]. The 10-year Zutphen study on Dutch middle-aged men showed no link between PP and dietary protein intake [26]. Conversely, a study of younger (< 30 years) white and Black South Africans revealed that despite lower total and animal protein intake among Blacks, they had higher serum protein levels [27]. The authors suggested this could stem from heightened protein catabolism or enhanced endogenous amino acid biosynthesis, potentially increasing collagen to mitigate early vascular aging risk. Given our older hypertensive and H-type participants, they might experience elevated PP from higher protein consumption. However, in individuals with normal BP, increased animal protein intake seemingly did not adversely affect arterial function.

MUFA consumption was positively associated with arterial function in all BP sub-groups, particularly in normal and H-type hypertension. The negative correlations between SFA intake and cr-PWV may stem from MUFAs and SFAs collinearity, as adjusting for MUFAs intake weakened these associations. Despite mixed evidence on MUFAs and vascular health, beneficial impacts on serum lipids, BP, and E-selectin suggest a lower CVD risk [28]. Further intervention studies are essential to substantiate MUFA intake recommendations and elucidate underlying mechanisms.

### Hcy categories, daily dietary intake and markers of cardiovascular function

Hcy measurements are more invasive and elective than recording BP but should be regarded as a necessary step in the approach to personalized nutrition, particularly in cardiovascular health. Incorporating Hcy screening into hypertension guidelines could differentiate between regular and H-type hypertension for targeted treatment.

Biotin, known for immunoregulatory properties affecting proinflammatory cytokine expression, which in turn induces ICAM-1 and VCAM-1 expression [29], showed positive correlations with Hcy in *CBS* T833C minor allele carriers [10]. However, research to confirm a direct relationship between dietary biotin and ICAM-1 remains to be done. We revealed that the positive associations between ICAM-1 and biotin get stronger when accounting for age more so than sugar in those with normal Hcy concentrations. It seems that the association between ICAM-1 and fruit and vegetable intake in the same Hcy group was unaffected by either. Notably, biotin and fruit/vegetable consumers ingested more added sugar and significance diminished after adjusting for sugar interactions.

Fructose induces ICAM-1 expression by diminishing endothelial nitric oxide and depleting adenosine triphosphate, leading to vascular cell inflammation [30]. Habitually high intake of carbohydrates, simple sugars, and fructose in particular, contributes to insulin resistance, weight gain and increased BP [31]. Some studies speculate that insulin resistance also produces a drop in methionine transmethylation, hepatic Hcy trans-sulphuration and Hcy clearance, resulting in raised concentrations of circulating Hcy [32, 33]. In Black South Africans, higher socio-economic status improves micronutrient intake through better access to fruits, vegetables, pulses, nuts, and seeds [15, 34] but also raises added sugar and SFA consumption, heightening non-communicable disease risks like CVD [35]. We, therefore, advise an adequate intake of fruit and vegetables, but caution against the accompanying consumption of added sugar.

### Hcy-related genetic SNPs, daily dietary intake and markers of cardiovascular function and inflammation

Incorporating genomic testing into CVD and hypertension guidelines could enhance personalized nutrition strategies, emphasizing the role of Hcy-related genetic polymorphisms in dietary impacts on cardiovascular health, particularly for H-type hypertension sufferers.

The relationship between sugar, SFAs and fruit and vegetable intake could explain the disappearance of the inverse association between added sugar and cr-PWV in variant allele carriers, which was stronger than in those homozygous for the wildtype allele at the *MTHFR* 677 locus, after accounting for fruit and vegetable intake. It could also help clarify why, after adjusting for sugar intake, the folate and VCAM-1 relationships disappeared in those carrying the *MTR* 2756 minor A genotype. The same can be said for positive associations betweenVCAM-1 and *MTR* 2756 major allele carriers for vitamin B_6_ intake. Lastly, the intake of thiamine, a precursor of coenzymes in sugar and amino acid catabolism, led to higher cr-PWV in the TT carriers *CBS* T883C/ins68 with the interactions disappearing after adjusting for added sugar. It seems therefore that an improved micronutrient status should be accompanied by a balanced, prudent diet of lower added sugar and fructose intake.

cr-PWV was inversely associated with lower PUFA intake in those harboring the TT genotype of *CBS* T883C/ins68. A previous intervention study reported that daily supplementation of 4,000 mg of omega-3 for 12 weeks decreased carotid-femoral PWV in older, but not younger, healthy adults independent of the effects on BP or arterial wave reflections [36]. The mechanism by which omega-3 PUFAs influence endothelial function (PWV) is mediated by their integration into the phospholipids of biological membranes, so affecting their fluidity. This interaction leads omega-3 PUFAs to bring about their beneficial effects such as increased nitric oxide production and reduced expression of proinflammatory mediators [37]. Despite the study population’s unusually low intake of omega-3 PUFAs, the beneficial implications should not be disregarded. A longitudinal PURE-SA study also found an inverse relationship between omega-6 PUFAs, SBP, and DBP [38], suggesting protective effects may highlight omega-6 long-chain products’ role in physiological functions among those with limited omega-3 consumption. The inverse association between SFAs intake and cr-PWV in those harboring *MTR* 2756AA could possibly be explained by the accompanying PUFAs intake.

Energy consumption was positively associated with VCAM-1 in *CBS* T883C/ins68 minor allele carriers and *CBS* 9276 homozygote major allele carriers. However, after adjustment for total carbohydrate intake the interaction disappeared, hinting at a potential link between energy from carbohydrates. Data on the relationship between energy intake and VCAM-1 are scarce. The additional adjustment for total carbohydrate intake that negated the interaction could be explained by the release of VCAM-1 in a dose-dependent manner when energy from carbohydrates increased glucose levels, as illustrated in a previous in vitro experiment using different doses of glucose in human umbilical vein endothelial cells (HUVECs) [39].

Positive linear associations between plant protein intake and VCAM-1 were observed in those harboring homozygote major alleles for *CBS* 9276 and/or *MTR* 2756, associations that remained after adjustments. However, an inverse relationship was observed for total protein intake and the *MTR* 2756 major homozygote AA carriers, suggesting a beneficial relationship. This is supported by previous studies that reported on animal and plant protein intake that decreased VCAM-1 expression in both unstimulated and stimulated HUVECs [40] as well as vascular endothelial cells [41]. The type of protein and concomitant nutrients could contribute to some of the different outcomes observed here.

### Recommendations for precision nutrition

International nutrition and hypertension guidelines offer general recommendations to prevent and manage cardiovascular risk [18, 42]. Although most guidelines are proposed for application across the larger population, and do not currently account for Hcy or H-type hypertension, our personalised nutrition approach still supports and fits within the following general guidelines: (1) lowered sugar intake; (2) sufficient intake of fruit and vegetables; (3) moderate intake of animal products; and (4) an adequate intake of healthy fats. We found that added sugar intake should be limited in those with seemingly normal Hcy or those carrying the T allele at *MTHFR* 677, to avoid possible detrimental associations with vascular activation (ICAM-1) and vascular function (cr-PWV). Reducing energy and sugar intake benefits *CBS* T883C/ins68 minor allele and *CBS* 9276 GG homozygote carriers regarding endothelial activation. Our results endorse consuming fruits and vegetables for their vitamin content, highlighting vitamin B_12_’s role in DBP management and meeting the RDA for those with hypertension. Achieving optimal vitamin status, particularly through fruits and vegetables, supports arterial health. Animal products, high in cholesterol and SFAs, should be consumed sparingly to manage SBP and PP, with a particular focus on animal protein for H-type hypertensives. Carriers of the *MTR* 2756 AA and *CBS* 9276 GG genotypes, on the other hand, should be sensible when consuming plant protein – lower rather than higher consumption could be beneficial in terms of vascular-related inflammation and atherosclerosis. Lastly, our results support an adequate intake of healthy fats such as PUFAs and MUFAs. Sufficient consumption of PUFAs is advised for carriers of the TT genotype of *CBS* T883C/ins68, owing to their positive influence on cr-PWV. We encourage also the intake of MUFAs within the normal ranges for all BP groups to ensure optimal cr-PWV and arterial wall function. Furthermore, dietary management is an alternative treatment avenue that could be explored for those who are resistant to traditional hypertensive drugs.

### Limitations and strengths

The study’s large population allowed detection of changes in cardiovascular function and gene–diet interactions, but a larger sample could better account for low-frequency SNPs. Due to its cross-sectional design, only associations were observed without causal inference. Recall bias in dietary assessment via the QFFQ may lead to under- or overreporting. For more precise results, we suggest using biochemical vitamin measures to capture absorption, metabolism and bioavailability, which dietary questionnaires overlook. Future research should conduct dietary supplementation trials by genotype and BP sub-groups to test their effectiveness. Additional cardiovascular markers such as carotid-femoral PWV, a “gold standard” measure for arterial stiffness, and intima media thickness should also be included in future research.

## Conclusions

This study enhances our understanding of Hcy-related nutrigenetics and cardiovascular health in Black South Africans, a population where gene–diet interactions with Hcy, BP, and cardiovascular markers are underexplored. Our findings demonstrate that dietary intake is associated with Hcy levels, BP groupings, and specific CVD risk markers, particularly when considering SNPs. We identified significant differences between regular hypertension and H-type hypertension, with the latter group exhibiting higher alcohol intake and lower consumption of macronutrients and micronutrients. If the gene-diet interactions reported here are replicated in further studies, personalized nutrition strategies, informed by genetic profiles and dietary interactions (e.g., fatty acids and proteins), could hold promise for improving cardiovascular outcomes.

### Electronic supplementary material

Below is the link to the electronic supplementary material.

Supplementary Material 1

## Data Availability

The data that support the findings of this study are available upon reasonable request and with the permission of the Health Research Ethics Committee of the North-West University and the principal investigator of the PURE-SA-NW study, Prof. Cristian Ricci (cristian.ricci@nwu.ac.za) at the North-West University’s Africa Unit for Transdisciplinary Health Research.

## References

[1] Mortality and causes of death in South Africa. 2014. Findings from death notification/Statistics South Africa.

[2] Yuyun MF, Sliwa K, Kengne AP, Mocumbi AO, Bukhman G. Cardiovascular diseases in sub-saharan Africa compared to high-income countries: an epidemiological perspective. Global Heart. 2020;15(1):15. 10.5334/gh.403. 32489788 10.5334/gh.403 PMC7218780

[3] Yoruk A, Boulos PK, Bisognano JD. The state of hypertension in Sub-saharan Africa: review and commentary. Am J Hypertens. 2018;31:387–8. 10.1093/ajh/hpx196. 29136102 10.1093/ajh/hpx196

[4] Kandala N-B, Nnanatu CC, Dukhi N, Sewpaul R, Davids A, Reddy SP. Mapping the burden of hypertension in South Africa: a comparative analysis of the national 2012 SANHANES and the 2016 demographic and Health Survey. Int J Environ Res Public Health. 2021;18:5445. 10.3390/ijerph18105445. 34069668 10.3390/ijerph18105445 PMC8160950

[5] Smith AD, Refsum H. Homocysteine–from Disease Biomarker to Disease Prevention. J Intern Med. 2021;90:826–54. 10.1111/joim.13279. 10.1111/joim.13279 33660358

[6] Fan R, Zhang A, Zhong F. Association between homocysteine levels and all-cause mortality: a dose-response Meta-analysis of prospective studies. Sci Rep. 2017;7:1–9. 10.1038/s41598-017-05205-3. 28684797 10.1038/s41598-017-05205-3 PMC5500552

[7] Zhong F, Zhuang L, Wang Y, Ma Y. Homocysteine levels and risk of essential hypertension: a Meta-analysis of published epidemiological studies. Clin Exp Hypertens. 2017;39:160–7. 10.1080/10641963.2016.1226888. 28287885 10.1080/10641963.2016.1226888

[8] Chen L, Wang B, Wang J, Ban Q, Wu H, Song Y, Zhang J, Cao J, Zhou Z, Liu L, Cao T. Association between Serum Total Homocysteine and arterial stiffness in adults: A Community-based study. J Clin Hypertens. 2018;20:686–93. 10.1111/jch.13246. 10.1111/jch.13246 PMC8031326 29481715

[9] Schutte AE, Srinivasapura Venkateshmurthy N, Mohan S, Prabhakaran D. Hypertension in low-and Middle-Income Countries. Circ Res. 2021;128:808–26. 10.1161/CIRCRESAHA.120.318729. 33793340 10.1161/CIRCRESAHA.120.318729 PMC8091106

[10] Du Plessis JP, Melse-Boonstra A, Zandberg L, Nienaber-Rousseau C. Gene Interactions Observed with the HDL-C blood lipid, intakes of protein, Sugar and biotin in relation to circulating homocysteine concentrations in a Group of Black South africans. Mol Genet Metab Rep. 2020;22:e100556. 10.1016/j.ymgmr.2019.100556. 10.1016/j.ymgmr.2019.100556 PMC6938949 31908954

[11] Nienaber-Rousseau C. Dietary strategies to treat hyperhomocysteinaemia based on the Biochemistry of Homocysteine: a review. South Afr J Clin Nutr. 2014;27:93–100. 10.1080/16070658.2014.11734495. 10.1080/16070658.2014.11734495

[12] Nienaber-Rousseau C, Pisa PT, Venter CS, Ellis SM, Kruger A, Moss SJ, Melse-Boonstra A, Towers GW. Nutritional Genetics: the case of Alcohol and the Mthfr C677t polymorphism in relation to Homocysteine in a Black South African Population. J Nutrigenet Nutrigenomics. 2013;6:61–72. 10.1159/000348839. 23548740 10.1159/000348839

[13] Chen L, Li Q, Fang X, Wang X, Min J, Wang F. Dietary intake of Homocysteine metabolism-related B-Vitamins and the risk of stroke: a dose-response Meta-analysis of prospective studies. Adv Nutr. 2020;11:1510–28. 10.1093/advances/nmaa061. 32503038 10.1093/advances/nmaa061 PMC7666912

[14] McNulty H, Strain J, Hughes CF, Ward M, Riboflavin. Mthfr genotype and blood pressure: a Personalized Approach to Prevention and Treatment of Hypertension. Mol Aspects Med. 2017;53:2–9. 10.1016/j.mam.2016.10.002. 27720779 10.1016/j.mam.2016.10.002

[15] Teo K, Chow C, Vaz M, Rangarajan S, Yusuf S. The prospective Urban Rural Epidemiology (pure) study: examining the impact of Societal influences on Chronic Noncommunicable diseases in Low-, Middle-, and high-income countries. Am Heart J. 2009;158:1–7. 10.1016/j.ahj.2009.04.019. 19540385 10.1016/j.ahj.2009.04.019

[16] MacIntyre U, Venter C, Vorster H. A culture-sensitive quantitative food frequency questionnaire used in an African Population: 2. Relative validation by 7-Day weighed records and biomarkers. Public Health Nutr. 2001;4:63–71. 10.1079/PHN200041. 11315682 10.1079/PHN200041

[17] Marfell-Jones M, Stewart A, Olds T. Kinanthropometry Ix. 1st ed. New York, NY, USA: Taylor & Francis; 2006. pp. 11–128.

[18] Unger T, Borghi C, Charchar F, Khan NA, Poulter NR, Prabhakaran D, Ramirez A, Schlaich M, Stergiou GS, Tomaszewski M, Wainford RD. 2020 International Society of Hypertension Global Hypertension Practice Guidelines. Hypertens. 2020;75:1334–57. 10.1161/HYPERTENSIONAHA.120.15026. 10.1161/HYPERTENSIONAHA.120.15026 32370572

[19] Li J, Jiang S, Zhang Y, Tang G, Wang Y, Mao G, Li Z, Xu X, Wang B, Huo Y. H-type hypertension and risk of stroke in Chinese adults: a prospective, nested case–control study. J Transl Int Med. 2015;3:171–78. 10.1515/jtim-2015-0027. 27847909 10.1515/jtim-2015-0027 PMC4936453

[20] Friedewald WT, Levy RI, Fredrickson DS. Estimation of the concentration of Low-Density Lipoprotein Cholesterol in plasma, without Use of the Preparative Ultracentrifuge. Clin Chem. 1972;18:499–502. 10.1093/clinchem/18.6.499. 4337382 10.1093/clinchem/18.6.499

[21] Nienaber-Rousseau C, Ellis SM, Moss SJ, Melse-Boonstra A, Towers GW. Gene–environment and gene–gene interactions of specific Mthfr, Mtr and Cbs Gene Variants in Relation to Homocysteine in Black South africans. Gene. 2013;530:113–8. 10.1016/j.gene.2013.07.065. 23954866 10.1016/j.gene.2013.07.065

[22] Karger AB, Steffen BT, Nomura SO, Guan W, Garg PK, Szklo M, Budoff MJ, Tsai MY. Association between homocysteine and vascular calcification incidence, prevalence, and progression in the MESA cohort. J Am Heart Assoc. 2020, 9, e013934. 10.1161/JAHA.119.013934 PMC7033888 32013703

[23] Reference Values for Arterial Stiffness’ Collaboration. Determinants of pulse wave velocity in healthy people and in the presence of cardiovascular risk factors:‘establishing normal and reference values’. Eur Heart J. 2010;31:2338–50. 20530030 10.1093/eurheartj/ehq165 PMC2948201

[24] du Plessis JP, Lammertyn L, Schutte AE, Nienaber-Rousseau C. H-Type hypertension among Black South africans and the relationship between Homocysteine, its genetic determinants and estimates of vascular function. J Cardiovasc Dev Dis. 2022;9:447–63. 10.3390/jcdd9120447. 36547444 10.3390/jcdd9120447 PMC9783379

[25] Chiu HF, Venkatakrishnan K, Golovinskaia O, Wang CK. Impact of micronutrients on hypertension: evidence from clinical trials with a special focus on Meta-Analysis. Nutrients. 2021;13:588–607. 10.3390/nu13020588. 33578935 10.3390/nu13020588 PMC7916651

[26] Mertens E, Tielemans S, Soedamah-Muthu S, Geleijnse J. Pulse pressure trajectories in relation to Cardiovascular Mortality and Dietary protein intake: the Zutphen Study. Proc Nutr Soc. 2015;74:e346. 10.1017/S0029665115003936. 10.1017/S0029665115003936

[27] Mels CM, Delles C, Louw R, Schutte AE. Central systolic pressure and a nonessential amino acid Metabolomics Profile: the African prospective study on the early detection and identification of Cardiovascular Disease and Hypertension. J Hypertens. 2019;37:1157–66. 10.1097/HJH.0000000000002040. 30801385 10.1097/HJH.0000000000002040 PMC6513088

[28] Du Y, Taylor CG, Zahradka P. Modulation of endothelial cell responses and vascular function by dietary fatty acids. Nutr Rev. 2019;77:614–29. 10.1093/nutrit/nuz026. 31228246 10.1093/nutrit/nuz026

[29] Rodriguez-Melendez R, Zempleni J. Regulation of Gene expression by Biotin☆. J Nutr Biochem. 2003;12:680–90. 10.1016/j.jnutbio.2003.07.001. 10.1016/j.jnutbio.2003.07.001 14690760

[30] Glushakova O, Kosugi T, Roncal C, Mu W, Heinig M, Cirillo P, Sanchez-Lozada LG, Johnson RJ, Nakagawa T. Fructose induces the inflammatory molecule Icam-1 in endothelial cells. J Am Soc Nephrol. 2008;19:1712–20. 10.1681/ASN.2007121304. 18508964 10.1681/ASN.2007121304 PMC2518440

[31] Basciano H, Federico L, Adeli K, Fructose. Insulin resistance, and metabolic dyslipidemia. Nutr Metab. 2005;2:1–14. 10.1186/1743-7075-2-5. 10.1186/1743-7075-2-5 PMC552336 15723702

[32] Han N, Chae JW, Jeon J, Lee J, Back HM, Song B, Kwon KI, Kim SK, Yun HY. Prediction of methionine and homocysteine levels in Zucker Diabetic Fatty (Zdf) rats as a T2dm animal model after consumption of a methionine-rich Diet. Nutr Metab. 2018;15:14–22. 10.1186/s12986-018-0247-1. 10.1186/s12986-018-0247-1 PMC5807833 29449868

[33] Tessari P, Cecchet D, Vettore M, Coracina A, Puricelli L, Kiwanuka E. Decreased Homocysteine Trans-Sulfuration in Hypertension with Hyperhomocysteinemia: relationship with insulin resistance. J Clin Endocrinol Metab. 2017;103:56–63. 10.1210/jc.2017-01076. 10.1210/jc.2017-01076 29029082

[34] Vorster H, Kruger A, Venter C, Margetts B, Macintyre U. Cardiovascular Disease Risk factors and Socio-Economic position of africans in Transition: the Thusa Study: Cardiovascular Topics. Cardiovasc J Afr. 2007;18:282–9. 17957323 PMC3975539

[35] Casari S, Di Paola M, Banci E, Diallo S, Scarallo L, Renzo S, Gori A, Renzi S, Paci M, de Mast Q, Pecht T. Changing Dietary habits: the impact of urbanization and rising socio-economic status in families from Burkina Faso in Sub-saharan Africa. Nutrients. 2022;14:1782–800. 10.3390/nu14091782. 35565752 10.3390/nu14091782 PMC9104313

[36] Monahan KD, Feehan RP, Blaha C, McLaughlin DJ. Effect of Omega-3 polyunsaturated fatty acid supplementation on central arterial stiffness and arterial Wave reflections in Young and older healthy adults. Physiol Rep. 2015;3:e12438. 10.14814/phy2.12438. 26109192 10.14814/phy2.12438 PMC4510635

[37] Zanetti M, Grillo A, Losurdo P, Panizon E, Mearelli F, Cattin L, Barazzoni R, Carretta R. Omega-3 polyunsaturated fatty acids: Structural and Functional effects on the Vascular Wall. BioMed Res Int. 2015;2015:1–15. 10.1155/2015/791978. 10.1155/2015/791978 PMC4537737 26301252

[38] Zec MM, Schutte AE, Ricci C, Baumgartner J, Kruger IM, Smuts CM. Long-chain polyunsaturated fatty acids are Associated with blood pressure and hypertension over 10-Years in black South African adults undergoing nutritional transition. Foods. 2019;8:394–409. 10.3390/foods8090394. 31500169 10.3390/foods8090394 PMC6770669

[39] Videm V, Albrigtsen M. Soluble Icam-1 and Vcam‐1 as markers of endothelial activation. Scand J Immunol. 2008;67:523–31. 10.1111/j.1365-3083.2008.02029.x. 18363595 10.1111/j.1365-3083.2008.02029.x

[40] Da Silva MS, Bigo C, Barbier O, Rudkowska I. Whey protein hydrolysate and branched-chain amino acids downregulate inflammation-related genes in vascular endothelial cells. Nutr Res. 2017;38:43–51. 10.1016/j.nutres.2017.01.005. 28381353 10.1016/j.nutres.2017.01.005

[41] Burris RL, Ng HP, Nagarajan S. Soy protein inhibits inflammation-Induced Vcam-1 and inflammatory cytokine induction by inhibiting the Nf-Κb and akt signaling pathway in apolipoprotein E–Deficient mice. Eur J Nutr. 2014;53:135–48. 10.1007/s00394-013-0509-7. 23468309 10.1007/s00394-013-0509-7 PMC3929972

[42] Siervo M, Lara J, Chowdhury S, Ashor A, Oggioni C, Mathers JC. Effects of the Dietary Approach to stop hypertension (dash) Diet on Cardiovascular Risk factors: a systematic review and Meta-analysis. Br J Nutr. 2015;113:1–15. 10.1017/S0007114514003341. 25430608 10.1017/S0007114514003341

