# Supplementary material for: Homocysteine, blood pressure and gene–diet interactions in relation to vascular function measures of black South Africans
Source: Genes Nutr. 2024 Aug 1;19:14. doi: 10.1186/s12263-024-00751-8 (PMC11295634; doi:10.1186/s12263-024-00751-8)
Supplement: Supplementary file 1 — Supplementary Material 1 [file 12263_2024_751_MOESM1_ESM.docx]

**Supplementary Table 1: Primers, PCR profiles, and restriction enzymes used to determine the genotypes**

| **Gene/**  **Polymorphism** | **Primer sequence** | **Annealing temperature** | **Restriction enzyme** | **Fragment lengths** |
| --- | --- | --- | --- | --- |
| ***MTHFR* C677T** | Forward primer: 5’-TGA AGG AGA AGG TGT CTG CGG GA-3’ | 55.0˚C | *HinfI* | PCR product: 198 bp  Digested: 176 bp, 22 bp |
|  | Reverse primer: 5’-AGG ACG GTG CGG TGA GAG TG-3’ |  |  |  |
| ***MTR* A2756G** | Forward primer: 5’-GAA CAT CCC AAG CCC ACT GAG-3’ (own design) | 58.0˚C | *BsuRI* (HaeIII) | PCR product: 440 bp  Digested: 260 bp, 180 bp |
|  | Reverse primer: 5’-GAC ACT GAA GAC CTC TGA TTT GAA C-3’ |  |  |  |
| ***CBS* T833C, 844ins68, G9276A** | Forward primer: 5’-ATA GAA TAT CGA GGC ATG TCC AGG CG-3’ | 66.5˚C | *BseNI* (*BsrI*) | PCR product: 282 bp (non-insert allele), 350 bp (insert allele)  Digested:  T833C wild-type: 257 bp, 25 bp  T833C mutant type: 180 bp, 77 bp, 25 bp  G9276A mutant type: 225 bp, 32 bp, 25 bp  T833C and G9276A mutant type: 148 bp, 77 bp, 32 bp, 25 bp  Insert allele: 325 bp, 25 bp*; 248 bp, 77 bp, 25 bp; 293 bp, 32 bp, 25 bp*; 216 bp, 77 bp, 32 bp, 25 bp* |
|  | Reverse primer: 5’-TGG GGC CCA GGG TCA GCC AGG CTC C-3’ |  |  |  |

- *No natural existence*

Supplementary Table 2

Demographic and dietary intake characteristics of participants among homocysteine sub-groups

| **Variables** | | | **Median (25^th^–75^th^), mean (±SD) or n (%)** | | | | **Difference among groups**  ***p* value** |
| --- | --- | --- | --- | --- | --- | --- | --- |
|  |  |  | **Whole group**  **(*n* = 1,867)** | **Low Hcy**  **(< 7.0 µmol/l)**  **(*n* = 341; 18.3%)** | **Normal Hcy**  **(7.0**–**12.0 µmol/l)**  **(*n* = 1,057; 56.7%)** | **High Hcy**  **(> 12.0 µmol/l)**  **(*n* = 469; 25.0%)** |  |
| **Age (years)** | | | 48.0 (41.0–56.0) | 44.0 (40.0–51.0) | 48.0 (41.0–56.0) | 51.0 (44.0–60.0) | <0.0001 |
| **Sex, *n* (%)** | Male | | 705 | 71 (10.1) | 392 (55.6) | 242 (34.3) | <0.0001 |
|  | Female | | 1162 | 270 (23.2) | 665 (57.2) | 227 (19.6) |  |
| **Urbanization level, *n* (%)** | Urban | | 910 | 193 (21.2) | 526 (57.8) | 191 (21.0) | <0.0001 |
|  | Rural | | 957 | 148 (15.5) | 531 (55.5) | 278 (29.0) |  |
| **Tobacco use, *n*** | Current | | 73 | 10 (13.7) | 50 (68.5) | 13 (17.8) | 0.05 |
|  | Former | | 976 | 187 (19.2) | 525 (53.8) | 264 (27.0) |  |
|  | Never | | 809 | 143 (17.7) | 474 (58.6) | 192 (23.7) |  |
| **Anthropometrical markers** | Weight (kg) | | 60.2 (51.2–72.9) | 61.8 (52.2–75.5) | 60.3 (51.3–74.2) | 57.4 (50.5–67.6) | 0.001 |
|  | BMI (kg/m^2^) | | 23.0 (19.3–28.9) | 24.1 (19.9–30.7) | 23.3 (19.2 –29.0) | 21.8 (18.8–26.5) | <0.0001 |
|  | Waist circumference (cm) | | 77.45 (70.2–87.7) | 78.4 (69.7–89.2) | 77.4 (70.0–88.0) | 76.9 (70.8–84.9) | 0.35 |
|  | Hip circumference (cm) | | 93.13 (84.8–106) | 96.3 (86.1–109) | 93.8 (85.0–106) | 90.0 (83.8–101) | <0.0001 |
|  | Waist-to-hip ratio | | 0.83 (0.78–0.88) | 0.82 (0.76–0.87) | 0.83 (0.78–0.88) | 0.85 (0.80–0.89) | <0.0001 |
| **Biochemical markers** | HIV status, *n* (%) | Sero-negative | 1549 | 261 (16.8) | 872 (56.3) | 416 (26.9) | <0.0001^€^ |
|  |  | Sero-positive | 310 | 77 (24.8) | 183 (59.0) | 50 (16.2) |  |
|  |  | Status unknown | 7 | 3 (42.8) | 2 (28.6) | 2 (28.6) |  |
|  | TC (mmol/l) | | 4.82 (4.01–5.87) | 4.67 (3.85–5.72) | 4.89 (4.05–5.94) | 4.82 (40.6–5.88) | 0.08 |
|  | LDL-c (mmol/l) | | 2.77 (2.07–3.63) | 2.71 (2.08–3.60) | 2.85 (2.15–3.73) | 2.63 (1.91–3.52) | 0.02 |
|  | HDL-c (mmol/l) | | 1.42 (1.06–1.87) | 1.31 (0.97–1.73) | 1.39 (1.06–1.82) | 1.60 (1.15 –2.16) | <0.0001 |
|  | Triglycerides (mmol/l) | | 1.08 (0.82–1.55) | 1.09 (0.81–1.51) | 1.08 (0.82 –1.54) | 1.06 (0.81 –1.60) | 0.56 |
|  | Fasting glucose (mmol/l) | | 4.80 (4.30–5.30) | 4.80 (4.30–5.30) | 4.80 (4.30–5.30) | 4.80 (4.20–5.40) | 0.66 |
|  | HbA1c (%) | | 5.50 (5.30–5.80) | 5.50 (5.30–5.90) | 5.60 (5.30 –5.80) | 5.50 (5.20 –5.80) | 0.02 |
|  | GGT (µkat/l) | | 46.0 (29.7–88.0) | 39.0 (26.9–66.8) | 43.1 (29.3–73.1) | 63.3 (33.1–153) | <0.0001 |
|  | Hcy (µmol/l) | | 9.18 (7.50–12.1) | 6.04 (5.47–6.59) | 8.97 (8.03–10.17) | 14.6 (13.0–17.7) | <0.0001 |
|  | CRP (mg/l) | | 3.29 (0.96–9.34) | 3.16 (0.84–8.97) | 3.20 (0.95–9.29) | 3.62 (1.15 –9.87) | 0.15 |
| **Cardiovascular markers** | SBP (mmHg) | | 130 (116–147) | 126 (113–142) | 128 (116–145) | 138 (122 – 154) | <0.0001 |
|  | DBP (mmHg) | | 87.0 (78.0–97.0) | 86.0 (76.5–95.0) | 86.0 (77.0–95.0) | 90.0 (81.0 – 101) | <0.0001 |
|  | Pulse pressure (mmHg) | | 43.0 (35.0–53.0) | 41.0 (34.0–50.0) | 43.0 (36.0–53.0) | 46.0 (37.0–57.0) | <0.0001* |
|  | Heart rate (bpm) | | 72.0 (62.0–84.0) | 71.5 (62.0–84.0) | 70.0 (61.0–82.0) | 77.0 (65.5–88.0) | <0.0001 |
|  | cr-PWV (m/s) | | 10.9 (9.55–12.2) | 10.6 (9.01–11.8) | 10.8 (9.54–12.1) | 11.2 (10.1–12.8) | <0.0001 |
|  | ICAM-1 (ng/ml) | | 371 (234–507) | 305 (216–458) | 367 (227–502) | 447 (289–560) | 0.23 |
|  | VCAM-1 (ng/mL) | | 693 (390–1279) | 662 (326–1259) | 717 (391–1301) | 706 (479–1155) | 0.26 |
| **Daily dietary intake markers** | Energy (kJ) | | 7175 (5268–10001) | 7614 (5516–10528) | 7125 (5195–10025) | 6959 (5240–9570) | 0.17^#^ |
|  | Alcohol intake (g) | | 11.8 ± 27.7 | 8.14 ± 20.1 | 11.1 ± 22.3 | 17.2 ± 28.8 | <0.0001 |
|  | Protein (%TE) | | 11.6 (10.4–12.9) | 11.9 (10.7–13.1) | 11.6 (10.3–12.9) | 11.3 (10.3–12.6) | 0.02 |
|  | Protein intake (g) | | 49.27 (34.8–71.5) | 53.7 (35.5–74.6) | 47.9 (34.8–72.7) | 46.9 (34.0–65.5) | 0.06^#^ |
|  | Plant protein intake (g) | | 28.9 (20.9–38.4) | 29.8 (22.0–39.3) | 28.4 (20.4–37.9) | 28.6 (20.7–38.7) | 0.86 |
|  | Animal protein intake (g) | | 18.4 (10.1–30.6) | 22.3 (11.7–33.6) | 18.4 (10.2–30.7) | 15.8 (9.00–26.6) | <0.0001 |
|  | Carbohydrate (%TE) | | 60.3 (54.2–67.5) | 59.9 (53.9–66.9) | 60.2 (54.6–67.8) | 60.6 (53.7–67.3) | 0.52 |
|  | Added sugar (%TCHO) | | 15.2 (9.51–21.8) | 15.6 (10.6–22.0) | 15.6 (9.66–22.7) | 13.8 (8.38–19.5) | <0.0001 |
|  | Total fat (%TE) | | 22.5 (17.4–27.7) | 23.7 (18.8–29.9) | 22.8 (17.7–27.8) | 21.0 (15.6–26.4) | <0.0001 |
|  | SFAs (%TE) | | 5.29 (3.63–7.06) | 5.63 (4.16–7.50) | 5.41 (3.71–7.02) | 4.78 (3.02–6.65) | <0.001 |
|  | MUFAs (%TE) | | 5.77 (3.75–7.74) | 6.18 (4.12–8.18) | 5.89 (3.89 –7.70) | 5.08 (3.29–7.36) | 0.001 |
|  | PUFAs (%TE) | | 6.78 (5.06–8.60) | 6.90 (5.15–8.84) | 6.85 (5.13–8.60) | 6.48 (4.65–8.31) | 0.04* |
|  | Omega-6 FAs (g) | | 12.0 (6.98–18.5) | 13.3 (7.67–20.8) | 12.0 (7.75–18.3) | 10.9 (6.17–17.3) | 0.01 |
|  | Omega-3 FAs (g) | | 0.34 (0.20–0.52) | 0.37 (0.23–0.58) | 0.34 (0.20–0.52) | 0.30 (0.18–0.44) | <0.0001 |
|  | Cholesterol (mg) | | 150 (80.0–259) | 175 (87.5–289) | 147 (77.7–259) | 138 (78.5–229) | 0.02 |
|  | Dietary folate (μg) | | 356 (248–481) | 373 (261–488) | 350 (248–487) | 354 (238–474) | 0.21 |
|  | Dietary vitamin B_1_ (mg) (thiamine) | | 1.49 (1.09–2.05) | 1.56 (1.13–2.10) | 1.45 (1.07–2.04) | 1.54 (1.09–2.05) | 0.77 |
|  | Dietary vitamin B_2_ (mg) (riboflavin) | | 1.01 (0.65–1.63) | 1.09 (0.69–1.67) | 0.98 (0.64–1.60) | 0.97 (0.68–1.60) | 0.98 |
|  | Dietary biotin (μg) | | 30.2 (19.1–46.3) | 34.75 (20.5–50.6) | 29.6 (18.8–46.6) | 27.7 (18.4–42.5) | 0.02 |
|  | Dietary pantothenic acid (mg) | | 3.67 (2.21–4.97) | 3.67 (2.36–5.18) | 3.39 (2.18–5.08) | 2.97 (2.10–4.43) | 0.001 |
|  | Dietary vitamin B_3_ (mg) (niacin) | | 12.7 (8.88–18.6) | 13.0 (9.30–18.5) | 12.6 (8.72–18.7) | 12.5 (8.94–18.5) | 0.62 |
|  | Dietary vitamin B_6_ (mg) | | 1.29 (0.92–1.84) | 1.43 (0.98–1.97) | 1.25 (0.90–1.84) | 1.26 (0.89–1.73) | 0.02 |
|  | Dietary vitamin B_12_ (μg) | | 2.69 (1.23–5.07) | 3.33 (1.51–5.88) | 2.68 (1.18–4.97) | 2.33 (1.13–4.67) | <0.01 |
|  | Fruit and vegetables (g) | | 86.6 (51.9–154) | 95.4 (56.7–174) | 87.9 (52.9–152) | 79.6 (48.6–135) | 0.03 |
|  | Pulses, nuts and seeds (g) | | 4.29 (0.00–19.3) | 4.29 (0–21.43) | 5.00 (0.00–19.57) | 3.57 (0.00–16.6) | 0.41 |

BMI, body mass index; CRP, C-reactive protein; cr-PWV, carotid-radial pulse wave velocity; DBP, diastolic blood pressure; GGT, gamma glutamyl transferase; Hcy, homocysteine; HDL-c, high-density lipoprotein cholesterol; HbA1c, glycated hemoglobin; HIV, human immunodeficiency virus; HR, heart rate; ICAM-1, intercellular adhesion molecule 1; LDL-c, low-density lipoprotein cholesterol; MUFAs, monounsaturated fatty acids; PP, pulse pressure; PUFAs, polyunsaturated fatty acids; SFAs, saturated fatty acids; SBP, systolic blood pressure; VCAM-1, vascular cell adhesion molecule 1.

*n* (%) calculated across rows for categorical variables.

Categorical variables: Pearson chi-squared test.

Continuous variables: GLM adjusted for age, WHR, GGT.

^€^Hcy concentrations did not differ between those with or without HIV (*P* = 0.99).

*Lost significance after adjustment.

^#^Became significant after adjustment.

Supplementary Table 3

Correlations of Hcy with demographic, anthropometric, biochemical, CVD risk markers and diet

| **Variables** | **Model 1** | | **Model 2*** | |
| --- | --- | --- | --- | --- |
|  | ***r*** | ***p*** | ***r*** | ***p*** |
| Age | 0.28 | <0.0001 | 0.16 | <0.001 |
| **Anthropometrical markers** | | | | |
| BMI (kg/m^2^) | –0.13 | <0.0001 | –0.07 | 0.10 |
| Waist circumference (cm) | –0.03 | 0.24 | 0.03 | 0.57 |
| Hip circumference (cm) | –0.14 | <0.0001 | –0.05 | 0.31 |
| Waist-to-hip ratio | 0.17 | <0.0001 | 0.08 | 0.09 |
| **Biochemical markers** | | | | |
| TC (mmol/l) | 0.05 | 0.02 | 0.11 | 0.01 |
| LDL-c (mmol/l) | –0.05 | 0.03 | <0.001 | 0.99 |
| HDL-c (mmol/l) | 0.19 | <0.0001 | 0.21 | <0.0001 |
| Triglycerides (mmol/l) | 0.001 | 0.96 | –0.09 | 0.04 |
| Fasting glucose (mmol/l) | 0.002 | 0.92 | –0.02 | 0.58 |
| HbA1c (%) | –0.05 | 0.02 | –0.02 | 0.74 |
| GGT (µkat/l) | 0.24 | <0.0001 | 0.15 | <0.001 |
| CRP (mg/l) | 0.03 | 0.15 | 0.01 | 0.84 |
| **Cardiovascular markers** | | | | |
| SBP (mmHg) | 0.19 | <0.0001 | 0.04 | 0.33 |
| DBP (mmHg) | 0.16 | <0.0001 | 0.07 | 0.13 |
| Pulse pressure (mmHg) | 0.14 | <0.0001 | –0.01 | 0.89 |
| Heart rate (bpm) | 0.11 | <0.0001 | 0.09 | 0.04 |
| cr-PWV (m/s) | 0.19 | <0.0001 | 0.03 | 0.56 |
| ICAM-1 (ng/ml) | 0.23 | <0.0001 | 0.03 | 0.57 |
| VCAM-1 (ng/ml) | 0.04 | 0.35 | 0.06 | 0.16 |
| **Dietary intake** | | | | |
| Energy (kJ) | –0.05 | 0.04 | –0.12 | 0.01 |
| Alcohol intake (g) | 0.16 | <0.0001 | 0.11 | 0.01 |
| Protein (%TE) | –0.05 | 0.04 | –0.01 | 0.87 |
| Protein intake (g) | –0.05 | 0.02 | –0.11 | 0.01 |
| Plant protein intake (g) | -0.08 | 0.001 | –0.08 | 0.09 |
| Animal protein intake (g) | -0.02 | 0.29 | –0.09 | 0.04 |
| Carbohydrate (%TE) | –0.04 | 0.11 | –0.03 | 0.48 |
| Added sugar (%TCHO) | –0.10 | <0.01 | –0.16 | <0.0001 |
| Total fat (%TE) | –0.10 | <0.0001 | –0.07 | 0.02 |
| SFAs (%TE) | –0.10 | <0.0001 | –0.10 | 0.02 |
| MUFAs (%TE) | –0.08 | <0.001 | –0.04 | 0.32 |
| PUFAs (%TE) | –0.06 | <0.01 | –0.03 | 0.50 |
| Omega-6 FAs (g) | –0.09 | <0.001 | –0.11 | 0.01 |
| Omega-3 FAs (g) | –0.09 | <0.001 | –0.10 | 0.02 |
| Cholesterol (mg) | –0.07 | <0.01 | –0.08 | 0.09 |
| Dietary folate (μg) | –0.04 | 0.07 | –0.07 | 0.11 |
| Dietary vitamin B_1_ (mg) (thiamine) | –0.02 | 0.48 | –0.07 | 0.13 |
| Dietary vitamin B_2_ (mg) (riboflavin) | 0.003 | 0.90 | –0.09 | 0.05 |
| Dietary biotin (μg) | –0.08 | <0.01 | –0.10 | 0.02 |
| Dietary pantothenic acid (mg) | –0.08 | <0.001 | –0.09 | 0.05 |
| Dietary vitamin B_3_ (mg) (niacin) | –0.001 | 0.97 | –0.05 | 0.25 |
| Dietary vitamin B_6_ (mg) | –0.06 | <0.01 | –0.09 | 0.03 |
| Dietary vitamin B_12_ (μg) | –0.07 | <0.01 | –0.10 | 0.02 |
| Fruit and vegetables (g) | –0.07 | 0.01 | –0.07 | 0.11 |
| Pulses, nuts and seeds (g) | –0.04 | 0.10 | –0.05 | 0.20 |

BMI, body mass index; CRP, C-reactive protein; cr-PWV, carotid-radial pulse wave velocity; DBP, diastolic blood pressure; FA, fatty acid; GGT, gamma glutamyl transferase; HDL-c, high-density lipoprotein cholesterol; HbA1c, glycated hemoglobin; HR, heart rate; ICAM-1, intercellular adhesion molecule 1; LDL-c, low-density lipoprotein cholesterol; MUFAs, monounsaturated fatty acids; %TE, percentage total energy; %TCHO, percentage energy from carbohydrates; PUFAs, polyunsaturated fatty acids; PP, pulse pressure; SBP, systolic blood pressure; SFAs, saturated fatty acids; TC, total cholesterol; VCAM-1, vascular cell adhesion molecule1.

*Partial Spearman correlations in Model 2 were adjusted for age, WHR, HDL-c and GGT, except for those where collinearity was of concern; age was not adjusted for itself; anthropometrical markers not for WHR; blood lipids not for HDL-c and alcohol intake and GGT were not adjusted for GGT.
